# A case of hyperkeratosis lenticularis perstans (Flegel's disease): Clinical, dermatoscopic, and histological aspects. Treatment with combined UVA-UVB narrowband phototherapy

**DOI:** 10.1016/j.jdcr.2024.09.018

**Published:** 2024-10-10

**Authors:** Chiara Aurora Delrosso, Stefania Erra, Giorgio Delrosso

**Affiliations:** a Dipartimento di Medicina Traslazionale, Università degli Studi del Piemonte Orientale e Ospedale Maggiore della Carità, Novara, NO, Italia; b Laboratorio di Anatomia Patologica, Clinica S. Rita, Gruppo Policlinico di Monza, Vercelli, VC, Italia; c Già Direttore S.S, di Dermatologia e Venereologia Presidio Ospedaliero S, Rocco di Galliate Azienda Ospedaliero-Universitaria “Maggiore della Carità”, Novara, NO, Italia

**Keywords:** dermoscopy, Flegel's disease, histopathology, hyperkeratosis lenticularis perstans, phototherapy, UVA-UVB narrowband phototherapy

## Introduction

Lenticular hyperkeratosis perstans, also known as Flegel's disease (FD), is a rare skin disease that usually affects middle-aged people.^1^ It manifests as numerous reddish-brown keratotic papules with a prevalent localization on the lower limbs, in people of middle or advanced age. Cases have been described in which FD can affect other body areas or tend to progressively involve almost the entire integument.^2^

We describe the case of a 32-year-old patient, with peculiar clinical aspects, dermoscopical, and histopathological characteristics, as well as their complete remission after treatment with combined UVA-UVB narrowband phototherapy.

## Case report

Caucasian male patient, 32 years old. He denied familiarity with peculiar dermatoses. No familiarity with cutaneous melanoma or nonmelanoma skin tumors. No continuous medication intake; occasional use of Farmaci Anti-infiammatori Non Steroidei (non-steroidal anti-inflammatory drugs) (F.A.N.S.) No known allergies; blood tests were normal.

**Fig 1:**
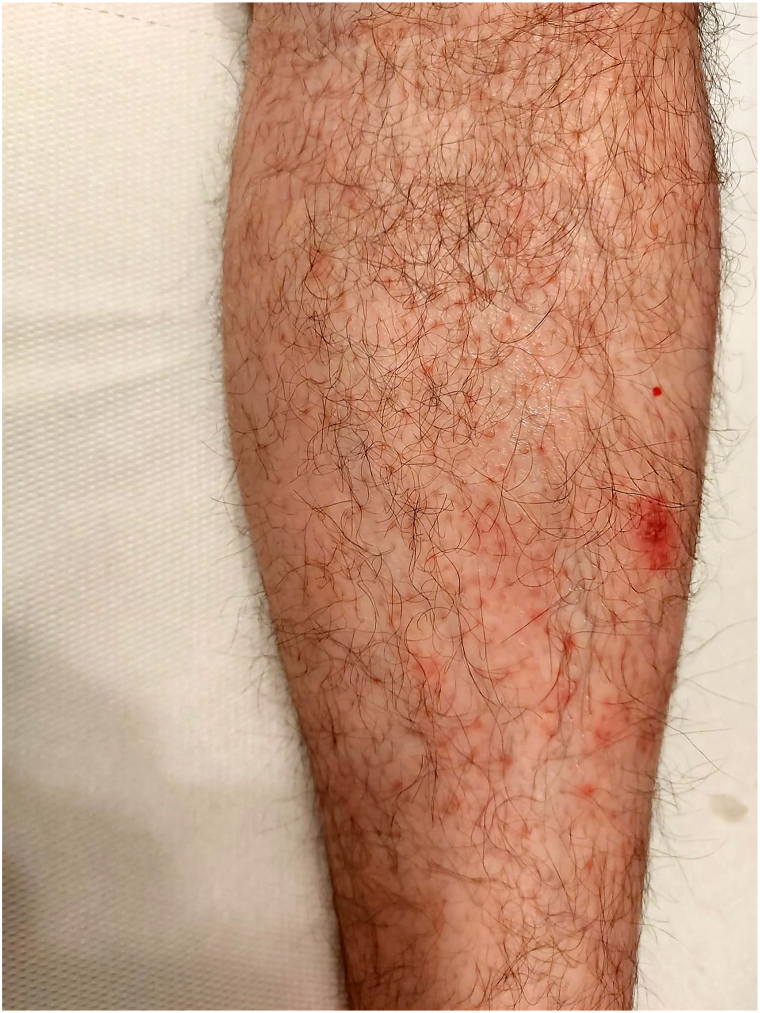
Lesion at first check-up, left leg (most significative area).

For about a year, the presence of reddish or brownish, lenticular papules was observed; almost asymptomatic, localized on the legs, mainly on the extensor surface (Fig 1), and with progressive involvement of the extensor surfaces of the forearms.

The patient did not present any particular pathologies; he was characterized as a phototype IV, according to Fitzpatrick’s scale, and he was in regular follow-up for diffuse nevomatosis; atypia at clinical, dermatoscopic, and videodermatoscopic evaluation was absent.

**Fig 2:**
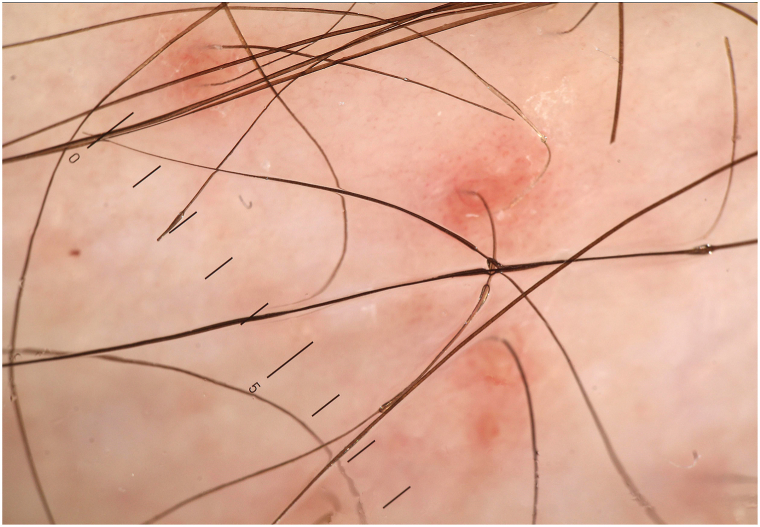
Dermatoscopic picture, left leg.

Dermatoscopy of the lesions highlighted reddish or brownish-red areas, with a non-atypical vascular pattern, characterized by ectasias of the apical component of the capillaries (since it is not a nail wall, that is, a reflection of a cutaneous plica, the capillary loop is visible only when viewed from the apex, within the dermal papilla; furthermore, the presence of ectasia of the postcapillary venules is also shown). This gives a brownish pseudoreticular appearance to the lesion with an erythematous base. There is a concomitant hyperkeratosis and a superficial desquamation between a furfuraceous and a pityriasic one (Fig 2).

**Fig 3:**
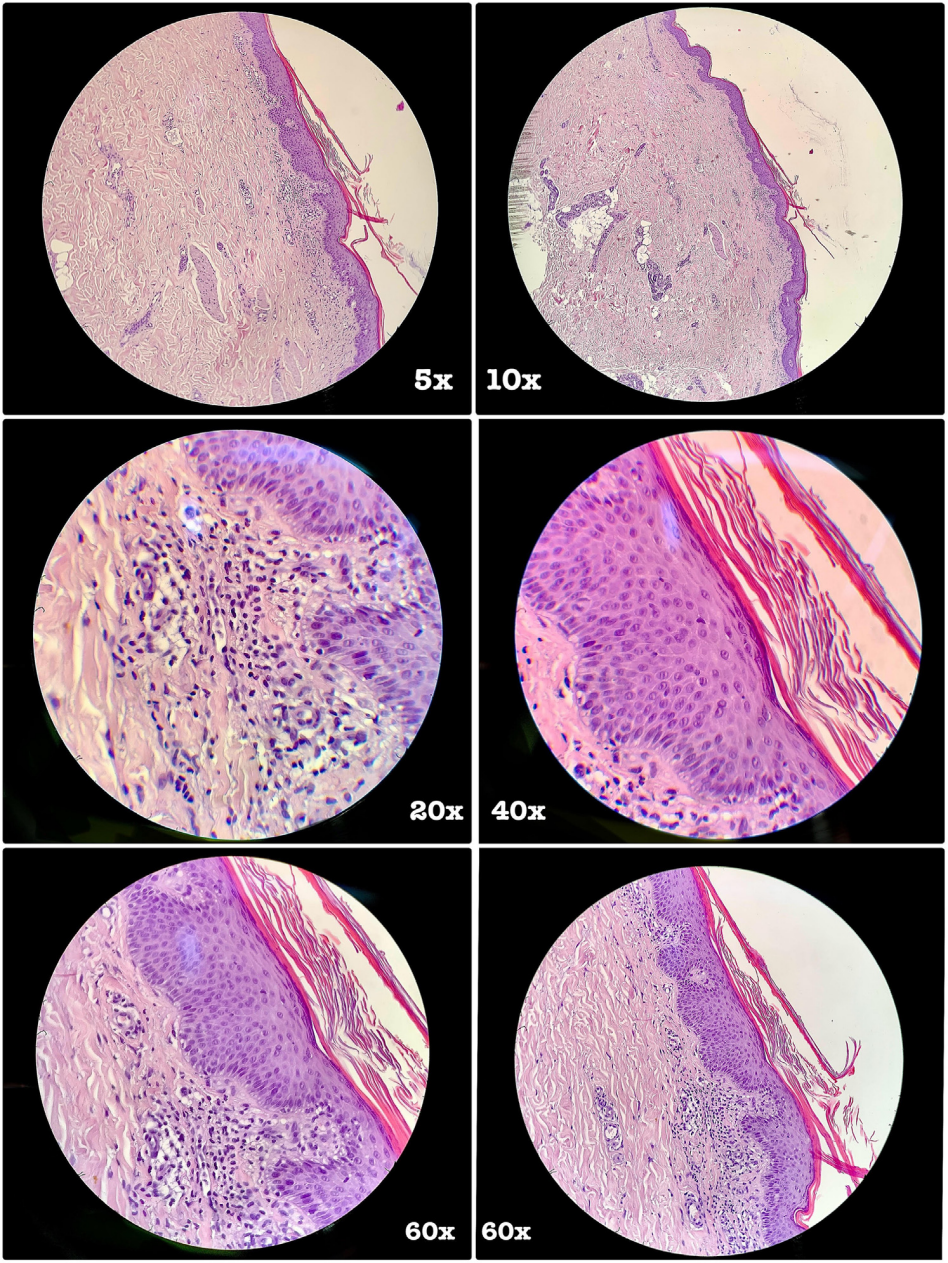
Histopathological pictures, respectively, with 5×, 10×, 20×, 40×, and 60× enlargements.

The histopathological examination of a lesion of the right leg highlighted an orthokeratotic-type hyperkeratosis and uniform acanthosis at the epidermal level. The papillary dermis presented edema and mild phlogistic lymphomonocytic infiltrate located interstitially and perivascularly. Furthermore, hyperplasia of the piloerector muscle bundles was noted (Fig 3).

Based on clinical, dermatoscopic, and histopathological data, we concluded for a form of generalized lenticular *hyperkeratosis perstans* (FD).

We started a treatment with combined narrowband UVA-UVB phototherapy.

## Discussion

FD is a rare skin disease; its most typical onset is between the fourth and fifth decade of life, with a prevalence in females by a ratio of 1.6:1.^1^ It is very rare in childhood.^3^ Its most typical localization is on the dorsa of the feet and on the lower third of the extensor surface of the legs.^4^ Atypical presentations such as in the periorbital axillary, palmar, and plantar regions have been described,^5^ as well as unilateral^6^ diffuse and generalized forms.^2^ Though rare, localization to the oral mucosa has also been described.

As to pathogenesis, while there are familiar cases that suggest the possibility of an autosomal dominant disease, no specific genes have been isolated to support this hypothesis.

Possible correlations with other pathologies have been reported, such as Lyme borreliosis, basal or squamous cell carcinomas, lung, digestive system, and bladder tumors.^1^ In our case, no pathologies of this nature were present.

With regard to differential diagnosis, Kyrle's disease should be considered (FD was, in fact, considered a variant of Kyrle's disease),^7^ as well as stuccokeratosis, Mibelli's porokeratosis,^8^ disseminated superficial actinic porokeratosis, and Hopf acrokeratosis verruciformis.

The treatments proposed and reported in the literature include, in addition to emollients, topical steroids, cream based on 5-fluorouracil, vitamin D3, and topical or systemic retinoids.^1^

Excisional ablative treatments, curettage, diathermocoagulation, dermabrasion, cryotherapy, or the use of CO2 laser can also be taken into consideration.

In the literature, we have found only one publication^9^ reporting the case of a patient with FD who responded to treatment with psoralens and UVA, that is, the association between the intake of psoralens orally and the subsequent exposure to UVA rays.

In our case, the dermatosis presented an elective localization on the legs, mainly the extensor surface, with progressive involvement of the extensor surface of the forearms.

The histopathological examination, in addition to the clinical, dermatoscopic, and evolutionary aspects, corroborated the diagnosis of FD.

We started a treatment with combined narrowband UVA-UVB phototherapy, two nonconsecutive sessions per week, with progressive increase up to a maximum dosage of 1.3J of UVB associated with 5J of UVA, for a total of 20 sessions. Each single session included a combination of narrowband UVB delivery (311 nanometers) through a cabin and subsequent exposure of the patient to a UVA delivery panel (peak emission at 352 nanometers); the treatment was associated with a topical therapy based on 20% urea and inositol.^10^

We achieved remission of the dermatosis, confirmed at the check-up after 2 months.

The treatment was well tolerated by the patient and no interruptions or reductions in the expected dosages were necessary.

At the 4-week checkup, a progressive reduction of the lenticular papules and of the reddish or brownish-red color were observed; in some areas of the upper limbs, the remission was almost total. Dermatoscopy confirmed the tendency towards remission of the dermatosis; the non-atypical vascular pattern remained, although reduced; the superficial desquamation between the furfuraceous and the pityriasem was clearly reduced or absent.

**Fig 4:**
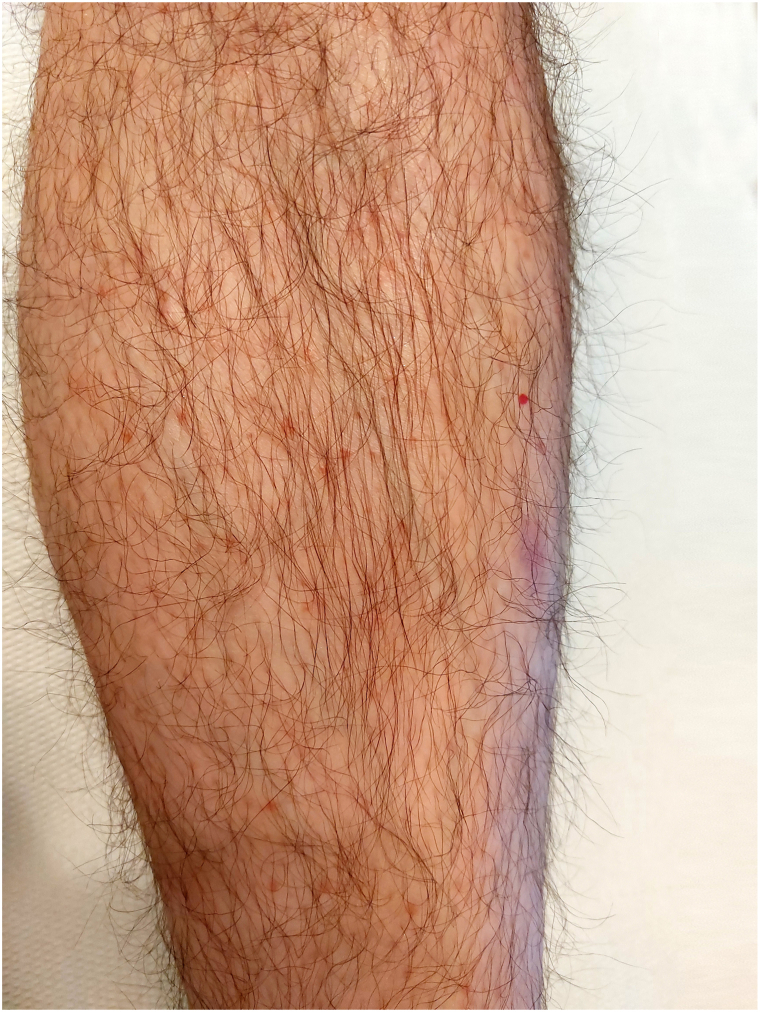
10 weeks check-up, left leg.

At the 10-week checkup (Fig 4), a complete remission of the lenticular papules as well as the reddish or brownish-red color were observed. Dermatoscopy confirmed the remission of the dermatosis.

## Conflicts of interest

None disclosed.
